# Changes in urbanicity and household availability of and proximity to food vendors from 2004 to 2020 in a rural district of northwestern Bangladesh

**DOI:** 10.1016/j.healthplace.2024.103374

**Published:** 2024-11

**Authors:** Alexandra L. Bellows, Amanda C. Palmer, Frank Curriero, Andrew L. Thorne-Lyman, Abu Ahmed Shamim, Saijuddin Shaikh, Rezwanul Haque, Hasmot Ali, Jonathon D. Sugimoto, Parul Christian, Keith P. West, Alain B. Labrique

**Affiliations:** a Department of International Health, Johns Hopkins Bloomberg School of Public Health, Baltimore, MD, USA; b Department of Epidemiology, Johns Hopkins Bloomberg School of Public Health, Baltimore, MD, USA; c J.B. Grant School of Public Health, BRAC University, Dhaka, Bangladesh; d Centre for Health Research and Development, Society for Applied Sciences, Delhi, India; e JiVitA Maternal and Child Health and Nutrition Research Project, Gaibandha, Bangladesh; f Seattle Epidemiologic Research and Information Center, Cooperative Studies Program, Office of Research and Development, United States Department of Veterans Affairs, Seattle, WA, USA; g Department of Epidemiology, University of Washington, Seattle, WA, USA; h Vaccine and Infectious Disease Division, Fred Hutchinson Cancer Research Center, Seattle, WA, USA

**Keywords:** Food environment, Nutrition transition, Bangladesh, Markets, Urbanicity

## Abstract

**Background:**

The nutrition transition underway in South Asia is likely mediated by changes to the food environment. Yet, few studies have been conducted in rural areas of South Asia to describe how the food environment has changed.

**Objective:**

This analysis assessed changes in household availability of and proximity to markets, grocery shops, and tea shops over a 16-year time period in Gaibandha, Bangladesh.

**Methods:**

We analyzed household demographic and geospatial data collected at 3 time points from 2004 to 2020 in a contiguous rural area (435 km^2^). We defined availability as number of food vendors within 400- and 1600-m radius of households and proximity as distance to nearest vendor. We used linear and Poisson models to estimate associations between household socioeconomic status (SES) and food vendor availability and proximity. We used multi-level models to conduct similar analyses for community-level urbanicity.

**Results:**

From 2004 to 2020, the numbers of markets, grocery shops and tea shops increased by 21%, 66% and 270%, respectively. Food vendor proximity did not change by household SES, but less urban households witnessed larger increases in proximity to markets (p for interaction<0.001) and tea shops (p for interaction<0.001) over time. Grocery shop and tea shop availability was initially higher and increased more over time for households in higher urbanicity areas (p for interaction<0.001).

**Conclusion:**

Over a 16-year period, this rural area of Bangladesh became more urbanized, increasing the availability of and proximity to markets, grocery shops, and tea shops. Further research is needed to see how these changes impact rural residents’ intake and nutritional status.

## Highlights

• As urbanicity increases over time, the food system in rural Bangladesh is transforming.
• The availability and proximity of markets, grocery shops, and tea shops increased in the study area in rural Bangladesh.
• Households living in less urban areas were more likely to see greater increases in market and tea shop proximity over time.
• Grocery shop and tea shop availability were higher in more urban areas and significantly increased over time in those areas.

## 1 Introduction

The nutrition transition is defined as changes in dietary and physical activity patterns driven by increased urbanization, globalization, and economic development (Popkin, 1993). In many countries, the consumption of saturated fat, vegetable oils, simple carbohydrates, added sugar, animal-source foods, and ultra-processed foods is increasing (Monteiro et al., 2013; Popkin, 2001). These changing dietary patterns are associated with the rising prevalences of overweight, obesity, and noncommunicable diseases (Hawkes et al., 2017). In certain contexts, this transition occurring alongside epidemiologic and demographic transitions can lead to a phenomenon known as the double burden of malnutrition, where the prevalence of overnutrition rises while the prevalence of undernutrition remains high (Popkin et al., 2020). Of particular concern are the rising prevalences of overweight and obesity in rural areas in South Asia and Sub-Saharan Africa (NCD Risk Factor Collaboration (NCD-RisC), 2019). Traditionally, in many countries in these regions, overweight and obesity were believed to be predominately associated with urban environments and lifestyles. However, food systems—the complex networks of people and processes that involve the production, processing, aggregation, distribution, purchasing, consumption, and waste of food (Parsons et al., 2019)—are now providing rural communities with increased access to a greater diversity of food, including less healthy food options (Popkin, 2014).

The food environment, where consumers interact with the food system, is defined by the availability, affordability, product properties (e.g., level of processing), marketing of food, and vendor properties (e.g., availability and proximity of different food vendors) (Downs et al., 2020; Herforth and Ahmed, 2015; Turner et al., 2018). It encompasses the places where people obtain their food and the types of food accessible within a person's normal routine (Turner et al., 2018). The food environment influences consumer behavior and dietary intake by guiding the types of food procured and consumed (Global Panel on Agriculture and Food Systems for Nutrition, 2016; HLPE, 2017; Turner et al., 2018). The majority of research on the food environment and its relationship to nutrition and health outcomes has been conducted in the Global North. Food environment results from these contexts may not be transferable to other settings because of the significant differences in food systems and aspects of the food environment (Downs et al., 2020; Turner et al., 2019). In South Asia, food environment typologies may be more diverse with both formal and informal markets and vendors partly due to the degree of urbanization (Downs et al., 2020, 2022). For countries such as Bangladesh, little research has been done to describe how the food environment, particularly the availability of and proximity to different food vendors, has changed over time in rural areas.

Bangladesh is in the early stages of the nutrition transition (Jaacks et al., 2019; Khan and Talukder, 2013). The country is experiencing rapid economic growth and increased urbanization along with low, but rising, prevalences of overweight and obesity (Biswas et al., 2017; Jaacks et al., 2019; Khan and Talukder, 2013). Notably, the average BMI is increasing more rapidly in rural versus rural areas of the country (NCD Risk Factor Collaboration (NCD-RisC), 2019). The dichotomization of urban/rural is likely to obscure changes in rural areas that may have important implications for food systems. Furthermore, Bangladesh is one of the most densely populated countries, with an average population density of 1252 people per sq km (Ritchie and Mathieu, 2019). Even in traditionally rural areas, the relative population density may have influences on the food environment. Assessing changes in the food environment by household wealth and community urbanicity level—defined as degree of urbanization, based on characteristics of economic activity, built environment, and population density—in a rural context may provide important information on how drivers of the nutrition transition influence the rural food environment.

This analysis aims to assess changes in urbanicity and household availability (number of food vendors in a defined radius of household) and proximity (distance to nearest food vendor) of food vendors over a 16-year time period (2004 to 2020) in a rural district of northwestern Bangladesh. Our hypotheses are that: 1) community-level, average urbanicity increases over time; 2) availability of and proximity to food vendors increases over time, with greater positive change for households with higher socioeconomic status; 3) urbanicity is associated with changes in availability of and proximity to different food vendors.

## 2 Methods

### 2.1 Setting

**Fig. 1:**
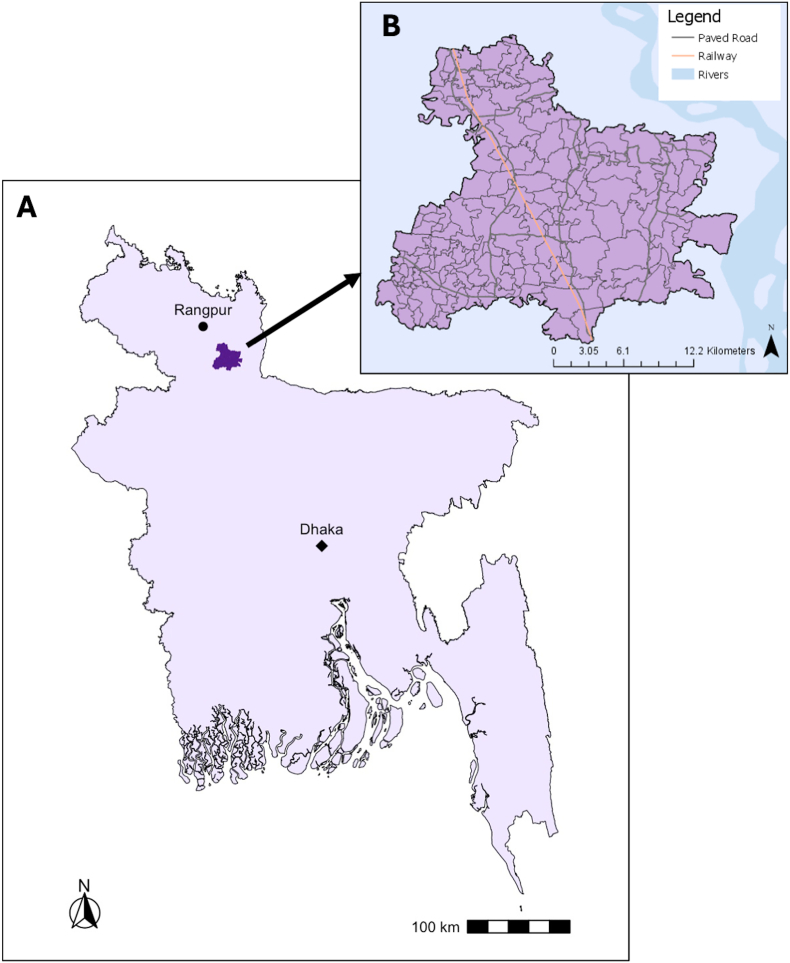
Map of JiVitA Study Area (2008–2019) Fig. 1A presents a map of Bangladesh with the JiVitA study site boundaries highlighted in dark purple. Fig. 1B is a map of JiVitA study site with mauza boundaries, paved roads, and railways.

Our analysis draws on data from the JiVitA Maternal and Child Health and Nutrition research site, which was established by researchers from Johns Hopkins University in 2000 (Fig. 1). JiVitA has been the site of multiple cluster-randomized community trials and observational studies focused on maternal and child health and nutrition (Labrique et al., 2011; West et al., 2011, 2014). The research site is approximately 435 km^2^, with a population of over 650,000 individuals (Sugimoto et al., 2007). The site comprised 19 rural unions in Gaibandha and Rangpur Districts in northwestern Bangladesh (Labrique et al., 2011). The 19 unions include 148 mauzas, an administrative unit synonymous with village or gram level. Unions were further divided into sectors (n = 596) by the research team to serve as units of randomization for JiVitA trials. This site was purposefully selected to reflect the infrastructural, agricultural, ecological, demographic, health and nutritional characteristics of rural Bangladesh, and has revealed results that have been found generalizable to rural life in the country and region (Sugimoto et al., 2007).

### 2.2 Subjects

#### 2.2.1 Household demographic data collection

This analysis utilizes data from baseline household surveys collected as part of three cluster randomized controlled trials: JiVitA-1 (August 2001–November 2006), JiVitA-3 (January 2008–September 2011), and mCARE-II (July 2016–May 2019) (Labrique et al., 2011; West et al., 2011, 2014). The size of the JiVitA research site was reduced after the completion of JiVitA-3.

Therefore, we restricted analyses of geospatial and survey data to 566 of the original 596 JiVitA sectors. Baseline characteristics of JiVitA-1 and JiVitA-3 trial participants have been previously published (West et al., 2011, 2014). All three trials used a pregnancy surveillance system, which identified all households in the area that included a married woman of reproductive age. Women were eligible for enrollment in JiVitA-1, JiVitA-3, and mCARE-II if households had a married woman of reproductive age residing in the household, the woman of reproductive age had a pregnancy during the trial period, and the household consented to be a part of the trial. Study participants provided informed consent. The study protocols for each trial were approved by the Johns Hopkins Bloomberg School of Public Health Institutional Review Board, Baltimore, Maryland, and the Bangladesh Medical Research Council, Dhaka, Bangladesh. Each trial was registered on clinicaltrials.gov (JiVitA-1: NCT00198822; JiVitA-3: NCT00860470; mCARE-II: NCT02909179).

After women provided consent, trained interviewers visited enrolled women and collected information on household demographic data such as home characteristics, household assets, adult occupations, and educational attainment. In the case where multiple women from the same household were enrolled in the same trial, household demographic information was collected more than once at each time of enrollment. For this analysis, we selected the demographic data from the observation closest to the date of landmark geospatial data collection.

#### 2.2.2 Geospatial data collection

The JiVitA geographic information system (GIS) team conducted a comprehensive geospatial survey of landmarks of interest and households in the study area starting in September 2003 and continuously updated that database until 2012. Further information on the JiVitA site geospatial survey and methods have been published elsewhere (Sugimoto et al., 2007). After 2012, global positioning survey (GPS) data were collected only for households in the JiVitA study area. We carried out an additional landmark GPS survey in December 2020. Trained field staff collected GPS coordinates of landmarks of interest using tablets with GPS capabilities. Landmarks included markets (a group of five or more shops that are open daily); tea shops (a vendor which sold hot tea (primary purpose), had a place for customers to sit while consuming tea, and did not serve rice or other cooked/prepared foods but may sell pre-packaged foods (e.g., biscuits, cake)); grocery shops (a shop which sells groceries such as soap, cigarettes, and packaged foods and is not part of a group of 5 or more other similar shops); community health clinics; and schools. We used geospatial data from Bangladesh Local Government Engineering (LGED) Road Database (Local Government Engineering Department (LGED), 2022) to identify paved roads in the research study site. All spatially referenced data were inputted into ArcPro (ESRI, 2022) for data management and spatial analysis.

### 2.3 Exposure assessment

#### 2.3.1 Urbanicity score

We constructed an urbanicity scale by adapting a scale developed for multi-country use in three settings: Ethiopia, India, and Peru (Novak et al., 2012). Our scale includes five domains: population density, economic activity, built environment, communication, and education. Each domain was weighted equally as has been done in other urbanicity scoring schemes (Dahly and Adair, 2007; Jones-Smith and Popkin, 2010; Novak et al., 2012). Scores were calculated at the mauza level (an administrative level in Bangladesh synonymous with village level) using the scoring system outlined in Supplemental Table 1.

To estimate population density, we used data from the WorldPop database (Tatem, 2017). Data were accessed for 2003, 2009, and 2018, which corresponded to the midpoint of the date ranges for baseline demographic surveys in the three trials. Population density in this database is estimated at the one sq km level (people/km^2^). To estimate mauza population density, we took the average population density within each mauza's administrative boundaries using the “Zonal Statistics as Table” tool in ArcPro, which summarizes values within specified boundaries.

For the economic activity domain, we calculated the percentage of the population involved in agriculture which was determined as the percentage of households where either head of household or participant reported working on their farm as their primary occupation (Novak et al., 2012). Scores were based on the percentage of the population not involved in agriculture.

For the built environment domain, we calculated the percentage of households with electricity and flush toilets using data from each trial's household demographic survey. At the mauza-level, communities were categorized (yes/no) as having access to electricity if at least 10% of households in the community reported having electricity. The presence of a paved road was assessed using geospatial data from Bangladesh Local Government Engineering (LGED) Road Database (Local Government Engineering Department (LGED), 2022).

For the communication domain, we calculated the percentage of households with a television and the percentage of households with at least one mobile phone. We used data from each trial's household demographic survey to calculate each variable. In the JiVitA-1 demographic survey, mobile phone ownership was not collected. Therefore, each mauza receives zero points for this component in JiVitA-1.

For the education domain, we assessed the presence of a primary school, the presence of a secondary school, and the percentage of women with secondary education or higher within each mauza. The presence of a school within each mauza was determined using cross-sectional data from the research site GPS survey (JiVitA-1: October 2004, JiVitA-3: June 2009, and mCARE-II: December 2020). To the calculate percentage of women with secondary education, we used data from each trial's household demographic survey. Secondary education was defined as passing the secondary school certificate (SSC) or 11 years+ of schooling.

Overall urbanicity scores could range from 0 to 50. Each domain was weighted equally (10 points). We used factor analysis to ensure that the scale measured one latent construct (unidimensionality) (Slocum-Gori and Zumbo, 2011). To assess the internal consistency of our scale, we calculated corrected item-scale correlations.

Factor analysis showed the first factor to have an eigenvalue of 2.82 and subsequent factors to have values less than 0.85, suggesting one latent construct. The internal consistency of the scale was considered acceptable, with a Cronbach's alpha of 0.76 (>0.70 is considered satisfactory (Bland and Altman, 1997)), and corrected-item scale correlations for each domain had values greater than 0.40 (Netemeyer et al., 2003). Initially, we included diversity (variance in housing index) and health domains (presence of a community clinic and presence of a medicine shop) in the urbanicity score. However, tests of internal consistency indicated these items should be dropped to improve the score. Cronbach alpha for the initial overall score (urbanicity score including diversity and health domains) was 0.69, and corrected-item scale correlations for the diversity and health domains were less than 0.20.

#### 2.3.2 Socio-economic status

SES was characterized using a living standards index (LSI) and calculated using principal component analysis (PCA) from household assets and housing characteristics. Exploratory analysis was conducted to look at the variance of each household asset. Assets that did not have significant variability were excluded prior to conducting PCA. All variables were centered at zero and scaled to unit variance (Gunnsteinsson et al., 2010). Given that household assets may have had different relationships to wealth at different time points in each trial period, we calculated LSIs separately for each trial. For example, household access to electricity may have had a stronger relationship to relative household wealth in the JiVitA-1 trial than in the mCARE-II trial because access to electricity became more prevalent over time. We used simple imputation methods for households missing data on variables used to calculate the LSI (Gunnsteinsson et al., 2010). We categorized the LSI into three categories in each trial period: bottom 40%, middle 40%, and top 20% of households.

### 2.4 Outcome assessment

To characterize the food environment, we included food vendor GPS waypoints collected at three time points—October 2004, June 2009, and December 2020—and household GPS waypoints collected during the demographic survey. October 2004 corresponds to the completion of the GPS landmark survey during the JiVitA-1 field trial. June 2009 is the midpoint for the JiVitA-3 baseline demographic survey, and December 2020 was the closest time point to the mCARE-II survey (occurred 18 months after completion of the mCARE-II baseline demographic survey). Food vendors included in this analysis are markets, tea shops, and grocery shops. Food environment variables were calculated at the household level. Two components of the food environment are described below.

#### 2.4.1 Food vendor availability

Availability was defined as the density of different types of vendors within 400 m and 1600 m of households (Bivoltsis et al., 2018) with women of reproductive age enrolled in one of the three trials. These boundaries were chosen to reflect availability in the immediate proximity to households and availability at the village level (Baldock et al., 2018; Li et al., 2019).

#### 2.4.2 Food vendor proximity

Proximity to different types of vendors was defined as the distance to the nearest vendor type for households (Bivoltsis et al., 2018) with women of reproductive age enrolled in one of the three trials, which was measured by straight-line Euclidean distance.

These two measures are complimentary but distinct geospatial measures of the food environment. Availability may act as a proxy measure for food vendor choice while proximity is a proxy measure for travel time to nearest vendor (Bivoltsis et al., 2018).

#### 2.4.3 Statistical analysis

Households missing geospatial or demographic data were excluded from the analysis. Analysis was conducted using ArcGIS Pro 2.5 (ESRI, 2022) and The R Statistical Computing Environment version 4.2 (R Core Team, 2022). Exploratory analysis was conducted to check distributions for urbanicity, LSI, food vendor availability, and food vendor proximity measures. To assess trends in urbanicity, we created choropleth maps and compared descriptive statistics of urbanicity across mauzas at each time point.

Regression analysis was used to model household-level outcomes (food vendor availability and proximity) as a function of household-level SES and community-level urbanicity. Since similar variables were used to create both LSI and urbanicity scores, separate regression models were run for each exposure of interest. Linear regression was used for proximity (distance to food vendors) outcome variables, and Poisson regression was used for availability (number of food vendors) outcome variables. Negative binomial regression was used in place of Poisson regression if regression residuals exhibited overdispersion. To assess changes in food vendor availability and proximity over time by level of urbanicity, we divided urbanicity scores from the 1st time point (2004) into tertiles and included it in the model with an interaction with time. Since different households are included at the three different time points, time was included in all models as a fixed effect. Regression models with urbanicity scores were extended to be multi-level to account for nesting of households in mauza using random effects. Regression results from both bivariable (including only time) and multivariable (including time plus household or community-level exposures) models are presented. *P* < 0.05 was considered significant. Profile likelihood confidence interval methods were used to calculate 95% confidence intervals (CIs) for beta estimates using the “confint” command from the MASS package in R. To check for spatial autocorrelation in each model, we calculated standardized residuals at each time point and then performed a Moran's I test on these residuals.

## 3 Results

Baseline demographic data was available for 52979, 41111, and 24957 households in JiVitA-1 (2001–2006), JiVitA-3 (2008–2011), and mCARE-II trials (2016–2019). A total of 110,881 households were included in this analysis, with 47,710 households enrolled in the JiVitA-1 trial (2001–2006), 39,362 households enrolled in the JiVitA-3 trial (2008–2011), and 23,809 households enrolled in the mCARE-II trial (2016–2019). Geospatial data was not available for 5269 in JiVitA-1 trial, 1749 in JiVitA-3 trial, and 1148 and mCARE-II trial and were therefore excluded from this analysis. Community-level characteristics from each trial are summarized for each trial in Table 1.

**Table 1:** Summary statistics for components of the urbanicity score across all mauzas (n = 146), by trial cohort.

Domain	Variable	JiVitA-1	JiVitA-3	mCARE-II
		(2001–2006)	(2008–2011)	(2016-2019)
Demographic	Population density (people/km^2^),^a^*mean ± SD*	1181.0 ± 238.2	1260.8 ± 241.7	1352.8 ± 159.4
Economic Activity	% Population involved in agriculture,^b^*mean ± SD*	28.8 ± 7.8	21.7 ± 7.1	16.4 ± 7.6
Built Environment	% Households with electricity,^b^*mean* ± SD	12.7 ± 11.5	21.6 ± 14.7	70.2 ± 15.0
	% Households with flush toilet,^b^ *mean ± SD*	0.3 ± 0.5	0.1 ± 0.2	11.6 ± 11.2
	Presence of a paved road,^c^*n (%)*	77 (52.7%)	79 (54.1%)	95 (65.1%)
	Community electricity access,^b^ *n (%)*	74 (50.1%)	109 (74.6%)	146 (100%)
Communication	% Households with television,^b^*mean ± SD*	8.3 ± 4.7	17.2 ± 7.8	33.4 ± 12.1
	% Households with mobile phones,^b^*mean ± SD*	NA^d^	36.1 ± 6.2	96.7 ± 2.0
Education	% Women with secondary education,^b^*mean ± SD*	6.9 ± 2.9	7.7 ± 3.7	18.7 ± 6.4
	Presence of a primary school,^e^ *n (%)*	133 (91.1%)	133 (91.1%)	133 (91.1%)
	Presence of a secondary school,^e^*n (%)*	72 (49.3%)	72 (49.3%)	72 (49.3%)
**Urbanicity Score**	Mean (SD)	14.2 (3.1)	18.3 (3.1)	26.2 (2.9)

a Population density data from the WorldPop database (22). Data were accessed for 2003 (JiVitA-1), 2009 (JiVitA-3), and 2018 (mCARE-II), which corresponded to the midpoint of the date ranges for baseline demographic surveys in the three trials.

b Data from trial household demographic surveys. Data for JiVitA-1 were collected from 2001 to 2006 (n = 47,710). Data for JiVitA-3 were collected from 2008 to 2011 (n = 39,362). Data for mCARE-II were collected from 2016 to 2019 (n = 23,809).

c Data on paved roads are from Bangladesh Local Government Engineering (LGED) Road Database (20).

d Data on mobile phones were not available for JiVitA-1 trial.

e Data from study site GPS survey from October 2004 (JiVitA-1), June 2009 (JiVitA-3), and December 2020 (mCARE-II).

### 3.1 Urbanicity score

**Fig. 2:**
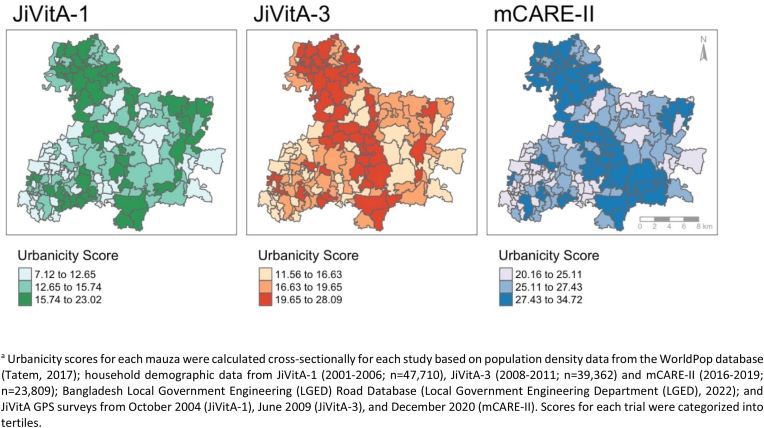
Spatial distribution of urbanicity scores,^a^ by trial cohort ^a^ Urbanicity scores for each mauza were calculated cross-sectionally for each study based on population density data from the WorldPop database (Tatem, 2017); household demographic data from JiVitA-1 (2001–2006; n = 47,710), JiVitA-3 (2008–2011; n = 39,362) and mCARE-II (2016–2019; n = 23,809); Bangladesh Local Government Engineering (LGED) Road Database (Local Government Engineering Department (LGED), 2022); and JiVitA GPS surveys from October 2004 (JiVitA-1), June 2009 (JiVitA-3), and December 2020 (mCARE-II). Scores for each trial were categorized into tertiles.

Urbanicity scores steadily increased over time and ranged from 7.1 in JiVitA-1 (2001–2006) to 34.7 in mCARE-II (2016–2019). Urbanicity scores increased uniformly over the three trials, with 63% (92/146) of mauzas categorized into the same urbanicity tertile in JiVitA-1 (2001–2006) and in mCARE-II (2016–2019) trials (Fig. 2).

Fig. 2 highlights higher urbanicity in the center and northern regions of the study site, which remained consistent over time. Mean urbanicity scores increased from 14.2 (SD: 3.09) in JiVitA-1 (2001–2006) to 26.2 (SD: 2.85) in mCARE-II (2016–2019) (Table 1). All components of the urbanicity score increased over time, with the greatest increase occurring within the communication domain as mobile phones became nearly ubiquitous in all households by mCARE-II (2016–2019) (Table 1).

### 3.2 Availability of and household proximity to food vendors

Over time, the number, availability, and proximity of food vendors increased for markets, grocery shops, and tea shops over time (Table 2, Table 3). The number of markets increased by 21% within 16 years while grocery shops and tea shops increased by 66% and 270%, respectively. Distance to nearest market decreased by 120 m, and on average, households had an additional market within 1600 m in 2020 compared to 2004. Distance to the nearest grocery shop for households decreased by 40 m, and availability of variety scores within 400 and 1600 m increased by 2 and 24 stores, respectively. We observed a decrease in the number of grocery shops from 2009 to 2020. Distance to the nearest tea shop for households decreased by over 500 m, and the average availability of tea shops within 1600 m of households increased by 7 shops (Table 2, Table 3).

**Table 2:** Summary statistics for proximity to and availability of food vendors^a^ over time.

	2004	2009	2020
**Markets**^b^			
Total (n)	223	230	270
Distance to Nearest Market (m) e	752.18 ± 414.50 f	722.57 ± 397.57	632.19 ± 343.46
Number of Markets within 400m g	0.35 ± 0.88	0.36 ± 0.87	0.44 ± 0.95
Number of Markets within 1600m g	3.95 ± 2.58	4.26 ± 2.71	5.04 ± 3.11
**Grocery Shops**^c^			
Total (n)	1870	3363	3102
Distance to Nearest Grocery Shop (m) e	216.93 ± 164.26	147.10 ± 114.47	177.60 ± 124.37
Number of Grocery Shops within 400m g	2.62 ± 1.92	5.04 ± 3.12	4.56 ± 3.18
Number of Grocery Shops within 1600m g	31.46 ± 9.88	60.99 ± 17.92	55.44 ± 16.64
**Tea Shops**^d^			
Total (n)	162	304	598
Distance to Nearest Tea Shop (m) e	1095.83 ± 720.06	786.22 ± 523.32	564.76 ± 394.03
Number of Tea Shops within 400m g	0.25 ± 0.67	0.50 ± 1.17	0.91 ± 1.49
Number of Tea Shops within 1600m g	3.02 ± 3.23	5.84 ± 5.60	10.96 ± 7.79

a Food vendors were identified using GPS data from October 2004 for the JiVitA-1 trial (2001–2006), June 2009 for the JiVitA-3 trial (2008–2011), and December 2020 for the mCARE-II trial (2016–2019).

b A group of five or more shops that are open daily.

c A shop which sells groceries such as soap, cigarettes, and packaged foods and is not part of a group of 5 or more other similar shops.

d A vendor which sold hot tea (primary purpose), had a place for customers to sit while consuming tea, and did not serve rice or other cooked/prepared foods but may sell pre-packaged foods (e.g., biscuits, cake).

e Distance to nearest food vendor (proximity) was measured straight line Euclidean distance from houses with enrolled women of reproductive age in JiVitA-1 (2004), JiVitA-3 (2009), mCARE-II (2020).

f Figures are mean ± SD.

g Density of food vendors (availability) within 400 m and 1600 m of households with enrolled women of reproductive age in JiVitA-1 (2004), JiVitA-3 (2009), mCARE-II (2020).

**Table 3:** Regression results assessing the change in the availability and proximity of food vendors over time. Incidence rate ratio (IRR) for negative binomial regression and coefficient slope estimates for linear models are presented along with their significance and 95% confidence interval (CI)**.**

Food Vendor	Change in Availability (400 m)^a^^,^^b^		Change in Availability (1600 m)^a^^,^^b^		Change in Proximity (m)^c^^,^^d^	
	IRR (95% CI)	p-value	IRR (95% CI)	p-value	Estimates (95% CI)	p-value
**Markets**^e^						
2004	NA^h^		*Ref*		*Ref*	
2009			1.08 (1.07, 1.09)	<0.001	−29.62 (−34.88, −24.36)	<0.001
2020			1.28 (1.26, 1.29)^i^	<0.001	−119.99 (−126.12, −113.86)	<0.001
**Grocery Shops**^f^						
2004	*Ref*		*Ref*		*Ref*	
2009	1.92 (1.91, 1.94)	<0.001	1.94 (1.93, 1.95)	<0.001	−69.83 (−71.70, −67.96)	<0.001
2020	1.74 (1.72, 1.76)	<0.001	1.76 (1.75, 1.77)	<0.001	−39.32 (−41.50, −37.15)	<0.001
**Tea Shops**^g^						
2004	*Ref*		*Ref*		*Ref*	
2009	1.99 (1.93, 2.05)	<0.001	1.94 (1.91, 1.96)	<0.001	−309.61 (−317.54, −301.67)	<0.001
2020	3.60 (3.48, 3.72)	<0.001	3.63 (3.58, 3.68)	<0.001	−531.07 (−540.32, −521.82)	<0.001

a Estimates from negative binomial regression models to account for over dispersion of outcome variables.

b Availability is defined as density of food vendors within 400 m and 1600 m of households with enrolled women of reproductive age in the JiVitA-1 trial (2001–2006; food vendors based on 2004 GPS data), JiVitA-3 (2008–2001; food vendors based on 2009 GPS data), mCARE-II (2016–2019; food vendors based on 2020 GPS data).

c Estimates from linear regression models.

d Proximity defined as distance to nearest food vendor was measured straight line Euclidean distance from households with enrolled women of reproductive age in JiVitA-1, JiVitA-3, and mCARE-II trials. Negative values indicate increased proximity.

e A group of five or more shops that are open daily.

f A shop which sells groceries such as soap, cigarettes, and packaged foods and is not part of a group of 5 or more other similar shops.

g A vendor which sold hot tea (primary purpose), had a place for customers to sit while consuming tea, and did not serve rice or other cooked/prepared foods but may sell pre-packaged foods (e.g., biscuits, cake).

h Markets within 400 m of households were not assessed as few households had a market within 400 m.

i Example interpretation: There is 28% increased risk of households in 2020 having 1 more market within 1600 m compared to households in 2004.

### 3.3 Availability of and household proximity to food vendors by SES

Households in the top 20% SES lived 20.6 m closer (95% CI: −28.6,-12.6; p-value: <0.001) to a market compared to households in the bottom 40% SES in 2004, but change in market proximity from 2004 to 2020 was not statistically significant by SES group (p-value for interaction >0.05). Availability of markets within 1600m of households nor change in availability differed by household SES status. Availability of and proximity to grocery shops also did not differ by household SES status. Households in the top 20% SES on average lived 35 m (95% CI: −45.6, −23.6; p-value: <0.001) closer to a tea shop compared to households in the bottom 40% SES in 2004, but change in tea shop proximity increased at a faster rate for those in the bottom 40% SES compared to those in the top 20% SES (p-value for interaction: <0.001). Availability of tea shops within 400m or 1600m of households did not differ by household SES status (Table 4).

**Table 4:** Regression results assessing the association between SES and the availability to and proximity of food vendors over time. Incidence rate ratio (IRR) for Poisson regression and coefficient estimates linear regression models are presented along with their significance and 95% confidence interval (CI).

	Availability (400 m)^a^^,^^b^		Availability (1600 m)^a^^,^^b^		Proximity (m)^c^^,^^d^	
	IRR (95% CI)	p-value	IRR (95% CI)	p-value	Beta (95% CI)	p-value
**Markets**^e^						
Intercept	NA^i^		3.67 (3.39, 3.96)	<0.001	747.95 (707.63, 788.26)	<0.001
Living Standard Index^h^						
Bottom 40%			*Ref*		*Ref*	
Middle 40%			1.00 (0.99, 1.01)	0.85	−6.42 (−13.02, 0.18)	0.06
Top 20%			1.01 (1.00, 1.02)	0.15	−20.62 (−28.61, −12.64)	<0.001
Year						
2004			*Ref*		*Ref*	
2009			1.08 (1.07, 1.10)	<0.001	−35.12 (−42.11, −28.14)	<0.001
2020			1.29 (1.27, 1.30)	<0.001	−126.77 (−134.87, −118.68)	<0.001
Interaction						
Middle 40%∗ 2009			1.00 (0.99, 1.02)	0.71	2.38 (−7.39, 12.15)	0.63
Top 20% ∗ 2009			0.99 (0.97, 1.01)	0.39	0.69 (−11.17, 12.55)	0.91
Middle 40% ∗ 2020			1.00 (0.99, 1.02)	0.87	2.41 (−8.97, 13.79)	0.68
Top 20% ∗ 2020			1.00 (0.98, 1.02)	0.95	11.45 (−2.38, 25.28)	0.10
**Grocery Shops**^f^						
Intercept	2.57 (2.45, 2.70)	<0.001	32.07 (30.83, 33.35)	<0.001	210.42 (203.28, 217.54)	<0.001
Living Standard Index						
Bottom 40%	*Ref*		*Ref*		*Ref*	
Middle 40%	1.02 (1.00, 1.03)	0.01	1.00 (1.00, 1.01)	<0.01	−3.27 (−6.01, −0.53)	0.02
Top 20%	1.01 (0.99, 1.02)	0.36	1.01 (1.00, 1.01)	<0.01	−3.34 (−6.66, −0.03)	0.05
Year						
2004	*Ref*		*Ref*		*Ref*	
2009	1.92 (1.90, 1.94)	<0.001	1.95 (1.94, 1.95)	<0.001	−73.24 (−76.14, −70.34)	<0.001
2020	1.75 (1.73, 1.77)	<0.001	1.79 (1.79, 1.80)	<0.001	−41.64 (−45.00, −38.28)	<0.001
Interaction						
Middle 40%∗ 2009	1.00 (0.99, 1.02)	0.78	1.00 (0.99, 1.00)	0.14	3.78 (−0.27, 7.84)	0.07
Top 20% ∗ 2009	1.01 (0.99, 1.03)	0.21	1.00 (0.99, 1.00)	0.45	6.68 (1.75, 11.61)	<0.01
Middle 40% ∗ 2020	0.98 (0.97, 1.00)	0.08	0.98 (0.98, 0.99)	<0.001	3.89 (−0.83, 8.62)	0.11
Top 20% ∗ 2020	1.00 (0.98, 1.03)	0.68	0.98 (0.97, 0.99)	<0.001	2.47 (−3.27, 8.21)	0.40
**Tea Shops**^g^						
Intercept	0.13 (0.10, 0.16)	<0.001	2.31 (2.05, 2.60)	<0.001	1078.65 (1016.04, 1141.27)	<0.001
Living Standard Index						
Bottom 40%	*Ref*		*Ref*		*Ref*	
Middle 40%	1.03 (0.99, 1.07)	0.22	1.01 (0.99, 1.02)	0.39	−15.21 (−24.23, −6.19)	<0.001
Top 20%	1.02 (0.97, 1.07)	0.42	1.00 (0.99, 1.01)	0.92	−34.73 (−45.65, −23.82)	<0.001
Year						
2004	*Ref*		*Ref*		*Ref*	
2009	2.05 (1.98, 2.13)	<0.001	1.95 (1.93, 1.97)	<0.001	−316.37 (−325.91, −306.83)	<0.001
2020	3.59 (3.47, 3.72)	<0.001	3.63 (3.59, 3.67)	<0.001	−556.53 (−567.59, −545.46)	<0.001
Interaction						
Middle 40%∗ 2009	0.94 (0.90, 0.99)	0.02	0.98 (0.96, 0.99)	<0.01	4.66 (−8.69, 18.01)	0.49
Top 20% ∗ 2009	0.94 (0.88, 1.00)	0.05	0.98 (0.96, 0.99)	0.01	17.02 (0.81, 33.23)	0.04
Middle 40% ∗ 2020	1.00 (0.95, 1.05)	0.88	1.02 (1.01, 1.04)	<0.001	22.34 (6.80, 37.89)	<0.01
Top 20% ∗ 2020	1.01 (0.95, 1.07)	0.72	1.03 (1.01, 1.05)	<0.001	43.92 (25.03, 62.82)	<0.001

a Estimates from multi-level Poisson regression models with a random effect for mauza included in all models.

b Availability is defined as density of food vendors within 400 m and 1600 m of households with enrolled women of reproductive age using GPS data from October 2004 for the JiVitA-1 trial (2001–2006), June 2009 for the JiVitA-3 trial (2008–2011), and December 2020 for the mCARE-II trial (2016–2019).

c Estimates from multi-level linear regression models with a random effect for mauza included in all models.

d Proximity defined as distance to nearest food vendor was measured straight line Euclidean distance from households with enrolled women of reproductive age in JiVitA-1, JiVitA-3, and mCARE-II (2020).

e A group of five or more shops that are open daily.

f A shop which sells groceries such as soap, cigarettes, and packaged foods and is not part of a group of 5 or more other similar shops.

g A vendor which sold hot tea (primary purpose), had a place for customers to sit while consuming tea, and did not serve rice or other cooked/prepared foods but may sell pre-packaged foods (e.g., biscuits, cake).

h A proxy for socioeconomic status and calculated using principal component analysis from household assets and housing characteristics.

i Markets within 400 m of households were not assessed as few households had a market within this radius.

### 3.4 Availability of and household proximity to food vendors by community-level urbanicity

In 2004, households living in communities in the highest urbanicity tertile lived an average of 193.2 m (95% CI: −287.2, −99.1; p-value: <0.001) closer to a market compared to households living in less urban communities, but change in market proximity over time was greater for households living in less urban communities (p-value for interaction: <0.001) (Table 5). Availability of markets within 1600m was also higher for households living in more urban areas in 2004 (incidence rate ratio (IRR): 1.34; 95% CI: 1.11, 1.61); p-value: <0.01), but change in availability of markets was slightly greater for households living in less urban communities. Households living in more urban communities were more likely to have higher availability of grocery shops within 1600m (IRR: 1.12; 95% CI: 1.02, 1.23) in 2004, with a slightly higher change in availability over time for households living in more urban communities (IRR: 1.06: 95% CI: 1.05, 1.06). Moreover, proximity of tea shops was greatest for households living in more urban areas in 2004, but change in proximity was greater for households in more rural areas over time. Households living in more urban communities were more likely to have higher availability of tea shops within 400m (IRR: 1.68; 95% CI: 1.02, 2.77) and 1600m (IRR: 1.20; 95% CI: 0.91, 1.59) in 2004, with higher change in availability of tea shops for more urban households over time (p-value for interaction <0.001) (Table 5).

**Table 5:** Regression results assessing the association between mauza-level urbanicity and the availability to and proximity of food vendors over time. Incidence rate ratio (IRR) for multi-level Poisson regression and coefficient estimates for multi-level linear regression models are presented along with their significance and 95% confidence interval (CI).

	Availability (400 m)^a^^,^^b^		Availability (1600 m)^a^^,^^b^		Proximity (m)^c^^,^^d^	
	IRR (95% CI)	p-value	IRR (95% CI)	p-value	Beta (95% CI)	p-value
**Markets**^e^						
Intercept	NA^f^		3.17 (2.78, 3.61)	<0.001	820.70 (754.49, 886.90)	<0.001
Urbanicity Tertile^e^						
1st			*Ref*		*Ref*	
2nd			1.16 (0.97, 1.40)	0.11	−43.64 (−137.21, 49.92)	0.36
3rd			1.34 (1.11, 1.61)	<0.01	−193.15 (−287.17, −99.14)	<0.001
Year						
2004			*Ref*		*Ref*	
2009			1.15 (1.14, 1.17)	<0.001	−63.47 (−71.92, −55.01)	<0.001
2020			1.32 (1.30, 1.34)	<0.001	−152.56 (−162.39, −142.73)	<0.001
Interaction						
Urbanicity 2 ∗ 2009			0.91 (0.90, 0.93)	<0.001	38.74 (27.51, 49.97)	<0.001
Urbanicity 3 ∗ 2009			0.93 (0.91, 0.94)	<0.001	41.50 (30.54, 52.45)	<0.001
Urbanicity 2 ∗ 2020			0.92 (0.90, 0.93)	<0.001	23.83 (10.73, 36.93)	<0.001
Urbanicity 3 ∗ 2020			1.01 (1.00, 1.03)	0.15	53.44 (40.69, 66.19)	<0.001
**Grocery Shops**^f^						
Intercept	2.55 (2.35, 2.77)	<0.001	30.38 (28.45, 32.45)	<0.001	211.78 (199.70, 223.86)	<0.001
Urbanicity Tertile						
1st	*Ref*		*Ref*		*Ref*	
2nd	0.98 (0.88, 1.10)	0.77	1.07 (0.97, 1.17)	0.17	−1.81 (−18.83, 15.22)	0.84
3rd	1.08 (0.96, 1.21)	0.18	1.12 (1.02, 1.23)	0.02	−7.52 (−24.60, 9.56)	0.39
Year						
2004	*Ref*		*Ref*		*Ref*	
2009	1.88 (1.85, 1.90)	<0.001	1.91 (1.90, 1.91)	<0.001	−79.20 (−82.71, −75.69)	<0.001
2020	1.62 (1.59, 1.64)	<0.001	1.70 (1.69, 1.71)	<0.001	−39.22 (−43.30, −35.14)	<0.001
Interaction						
Urbanicity 2 ∗ 2009	1.03 (1.01, 1.05)	<0.001	1.04 (1.03, 1.05)	<0.001	8.17 (3.50, 12.83)	<0.001
Urbanicity 3 ∗ 2009	1.03 (1.01, 1.05)	<0.001	1.02 (1.01, 1.02)	<0.001	15.58 (11.03, 20.12)	<0.001
Urbanicity 2 ∗ 2020	1.08 (1.06, 1.11)	<0.001	1.06 (1.05, 1.07)	<0.001	0.80 (−4.64, 6.24)	0.77
Urbanicity 3 ∗ 2020	1.12 (1.10, 1.14)	<0.001	1.06 (1.05, 1.06)	<0.001	−1.55 (−6.84, 3.75)	0.57
**Tea Shops**^**g**^						
Intercept	0.10 (0.07, 0.15)	<0.001	2.20 (1.82, 2.66)	<0.001	1160.63 (1054.19, 1267.06)	<0.001
Urbanicity Tertile						
1st	*Ref*		*Ref*		*Ref*	
2nd	1.20 (0.72, 2.03)	0.48	1.02 (0.78, 1.35)	0.87	−126.70 (−277.15, 23.76)	0.10
3rd	1.68 (1.02, 2.77)	0.04	1.20 (0.91, 1.59)	0.19	−158.72 (−309.92, −7.52)	0.04
Year						
2004	*Ref*		*Ref*		*Ref*	
2009	1.87 (1.79, 1.95)	<0.001	1.72 (1.70, 1.74)	<0.001	−300.82 (−312.35, −289.28)	<0.001
2020	2.96 (2.83, 3.09)	<0.001	3.14 (3.10, 3.18)	<0.001	−616.39 (−629.80, −602.97)	<0.001
Interaction						
Urbanicity 2 ∗ 2009	1.00 (0.94, 1.07)	0.93	1.07 (1.05, 1.09)	<0.001	−0.36 (−15.69, 14.98)	0.96
Urbanicity 3 ∗ 2009	1.13 (1.07, 1.20)	<0.001	1.23 (1.21, 1.25)	<0.001	−25.34 (−40.29, −10.39)	<0.001
Urbanicity 2 ∗ 2020	1.26 (1.19, 1.34)	<0.001	1.16 (1.14, 1.18)	<0.001	124.09 (106.21, 141.97)	<0.001
Urbanicity 3 ∗ 2020	1.33 (1.26, 1.40)	<0.001	1.29 (1.27, 1.31)	<0.001	91.31 (73.90, 108.71)	<0.001

a Estimates from multi-level Poisson regression models with a random effect for mauza included in all models.

b Availability is defined as density of food vendors within 400 m and 1600 m using GPS data from October 2004 for the JiVitA-1 trial (2001–2006), June 2009 for the JiVitA-3 trial (2008–2011), and December 2020 for the mCARE-II trial (2016–2019).

c Estimates from multi-level linear regression models with a random effect for mauza included in all models.

d Proximity defined as distance to nearest food vendor was measured straight line Euclidean distance from households with enrolled women of reproductive age in JiVitA-1, JiVitA-3, and mCARE-II.

e Urbanicity scores for each mauza were calculated based on population density data from the WorldPop database (22); household demographic data from JiVitA-1 (n = 47,710), Bangladesh Local Government Engineering (LGED) Road Database (20); and JiVitA GPS surveys from October 2004 (JiVitA-1).

f Markets within 400 m of households were not assessed as few households had a market within this radius.

## 4 Discussion

From 2004 to 2020, urbanicity steadily increased in an area considered to be traditionally rural in the context of Bangladesh. In combination with this increase in urbanicity, the number, availability, and proximity of markets, grocery shops, and tea shops increased throughout the JiVitA Research Site. Changes in food vendor proximity did not differ by household SES, but households living in less urban areas were more likely to see greater increases in market and tea shop proximity over time. On the other hand, grocery shop and tea shop availability were higher in more urban areas and there was a more significant increase in availability in those areas over time.

Our analysis provides evidence that the food system in rural Bangladesh is transforming. Interestingly, increases in urbanicity were relatively uniform over the study area, with all domains increasing over time. This may have important impacts on nutrition and health over time as higher urbanicity is associated with increased risk of diet-related risk factors and chronic diseases such as type 2 diabetes, cardiovascular disease, and stroke (Dahly and Adair, 2007; Vlahov, 2002). For example, in rural Uganda, participants living in villages with higher urbanicity scores were more likely to have lower physical activity, lower consumption of fruit, and higher body mass index (Riha et al., 2014). To our knowledge, only one other study has assessed the association between urbanicity and changes in the food environment over time. Using data from the China Health and Nutrition Survey, an analysis found results similar to ours (Wu et al., 2017). Higher urbanicity was associated with greater availability and proximity of supermarkets, free markets, fast-food restaurants, and other indoor restaurants. The study in China did not report if there were significant differences in changes in availability and proximity of food vendors over time by the level of urbanicity (Wu et al., 2017).

Our analysis addresses a critical gap in the literature as most studies assessing food environments in South Asia have been cross-sectional in design (Turner et al., 2019). Most longitudinal studies assessing changes in the food environment have been conducted in HIC settings (Caspi et al., 2012; Lytle and Sokol, 2017). For example, a study in New Zealand found that from 2005 to 2015 accessibility of both fast-food vendors and supermarkets increased over time, with households living in poorer neighborhoods having the largest increase in supermarket access (Hobbs et al., 2021). Moreover, a study in the United States found a rise in proximity and availability of supermarkets, convenience stores, and bakeries from 1971 to 2008. This study found households in poorer census tracts had a slightly greater increase in access to fast-food restaurants, but there was no effect measure modification by census tract poverty rates for supermarket access (James et al., 2017). While these two contexts are quite different from the setting of our research (i.e., supermarkets and modern fast-food restaurants are not yet present), we did find households with higher SES lived slightly closer to markets, but there was no difference in change in market proximity or availability by SES status over time. In addition, we found that households with lower SES had a greater change in tea shop proximity over time. While tea shops in Bangladesh and fast food vendors in the United States are quite different, both food vendors are more likely to sell less healthy food options. Tea shops frequently sell packaged and ultra-processed snacks in addition to tea (with sugar).

The significant increase in grocery shops and tea shops is particularly interesting and potentially worrisome. The prevalence of grocery shops nearly doubled from 2004 to 2020, while tea shop prevalence nearly tripled. Certain aspects of urbanization—such as growing road infrastructure, reduction in agriculture as primary employment, and increased education—may drive these changes. Growing road infrastructure may improve vendors' access to consumers and food suppliers, making these businesses more profitable (Vos and Cattaneo, 2020). In addition, increased education and income from non-agricultural activities are associated with improved household income and expenditures, allowing for households to have greater purchasing power for food sold in these types of stores (de Bruin et al., 2021; Nisbett et al., 2017). We did see a slight decrease in grocery shop prevalence from 2009 to 2020, which may indicate market saturation or a consequence of lockdowns and economic hardship due to the ongoing COVID-19 pandemic. Both grocery shops and tea shops are likely to sell processed, packaged, and ready-to-eat food; therefore, an increased prevalence of these vendors may increase availability and access to ultra-processed foods. Several observational studies and one randomized controlled trial have found a positive association between ultra-processed food consumption and the prevalence of overweight and obesity (Hall et al., 2019; Juul et al., 2018; Louzada et al., 2015; Silva et al., 2018). While the prevalence of overweight and obesity may still be relatively low in rural South Asia, body mass index is rising faster in rural areas than in urban areas (NCD Risk Factor Collaboration (NCD-RisC), 2019). A study assessing the relationship between the food environment and cardiovascular disease in peri-urban communities outside Hyderabad, India, found a significant but small association between higher density/availability of vendors selling processed foods and increases in BMI and waist circumference (Li et al., 2019). Similar to our analysis, this study also looked at two different definitions of availability: stores within 400m of a household and 1600m. Their results suggest a stronger association between an individual's immediate food environment (within 400m of a person's residence) and cardiovascular risk factors. We found that availability of both grocery shops and tea shops within 400m and 1600m of households increased over time, but there was no significant difference by either household SES status or community urbanicity score.

On the other hand, the increase in market access and availability over time may positively impact dietary diversity and food security. A nationally representative survey of rural households in Bangladesh (BIHS) found that households closer to markets are more likely to have higher household dietary diversity, a proxy measure of food security, and women's dietary diversity, a proxy measure of micronutrient deficiency (Islam et al., 2018). Studies in Tanzania, Malawi, Kenya, Indonesia, and Ethiopia also found that distance to local markets was inversely associated with dietary diversity for smallholder farmers (Koppmair et al., 2017; Madzorera et al., 2021; Sibhatu et al., 2015). A study among smallholder farmers in rural Pakistan found that distance to the market was associated with increased food insecurity (Ahmed et al., 2017). It is important to note that the average distance to market in the studies in Bangladesh and Pakistan were 1.7 km and 13 km, respectively, which is greater than the average distance to market in our study site, <1 km (Ahmed et al., 2017; Islam et al., 2018). The association between food security and market access may also be particularly important in this study's context as a previous analysis found a high prevalence of food insecurity, with 34% of households reporting moderate or severe household food insecurity (Na et al., 2016).

Studies assessing the relationship between market access and food security have generally been carried out among smallholder farmers (Ahmed et al., 2017; Jones, 2017; Koppmair et al., 2017; Sibhatu et al., 2015). The relationship between market access, dietary diversity, and food security may differ for farming households compared to non-farming households. Households not engaged in agriculture may rely more on market access and availability for food procurement compared to smallholder farmers who may supplement household food through home production. As areas urbanize, employment in agriculture typically declines due to an increase in non-agricultural employment opportunities and a decrease in land available for agricultural production (Abu Hatab et al., 2019; de Bruin et al., 2021). We see similar trends in our study where the majority of households did not rely on farming as their main source of income. Participation in agriculture within the JiVitA Research Site has declined from nearly 30% of households in 2004 to approximately 16% in 2018.

Our study has a number of limitations. First, data were not collected on food availability, food price, mode of transport, or other behaviours influencing access. Therefore, we relied exclusively on GPS coordinates of households and food vendors, which only describes households' proximity to and availability of food vendors but does not fully measure accessibility or give insight into food availability or affordability at each vendor. For example, in the United States, affordability of healthy foods had a stronger association with high diet quality and lower prevalence of obesity than proximity (Aggarwal et al., 2014; Drewnowski et al., 2012). Second, due to field constraints, geospatial data on food vendors was paused in 2012. The final round of geospatial data occurred approximately two years after the mean date of baseline data collection for the mCARE-II trial. In addition, data collection took place in December 2020 during the COVID-19 pandemic. Food vendors, particularly smaller shops, may have been forced to close due to economic constraints of lockdowns and social distancing measures. Therefore, the trend in grocery shops and tea shops growth may have been underestimated. Third, the development of the urbanicity score was constrained by previously collected baseline household demographics. Consequently, critical aspects of urbanicity may not have been accurately captured. For example, diversity is an important component of urbanicity scores (Cyril et al., 2013; Jones-Smith and Popkin, 2010; Novak et al., 2012), but we were unable to measure diversity in education because we collected educational attainment as a categorical variable. Within the communication domain, we included access to a television and mobile phone but lacked data on access to computers and the internet. Computer ownership and internet access may be important measures to distinguish differential communication access between communities. Moreover, to measure proximity, we calculated straight-line Euclidian distances from households which may not accurately capture the actual route people may use to travel to a food vendor. This is likely to underestimate the distance to nearest vendor and not take into account barriers and desirable routes (such as road and footpaths). Nevertheless, given the high density of households and food vendors in the study area, we believe that actual routes would be unlikely to differ significantly. Finally, these results may not be generalizable to other rural contexts due to Bangladesh's high population density, even in rural areas.

Despite these limitations, our analysis is among the first to provide evidence that the food environment is transforming in a rural South Asian setting. Urbanicity scores allow us to see heterogeneity in food vendor access and availability that would be lost if using a binary urban/rural definition (Cyril et al., 2013). In addition, this analysis demonstrates that researchers can leverage extant data from large field trials to assess changes in urbanicity and the food environment over time. Since the prevalence of overweight and obesity is rising along with the persistently high prevalence of food insecurity and undernutrition in rural areas of South Asia, it is crucial for researchers and policymakers to understand changes in the food environment. Measuring and monitoring these changes would allow for policymakers to identify potential targets for interventions, such as zoning laws or restrictions on unhealthy food promotion, to improve the food environment (Pineda et al., 2024). Further research is necessary to explore how changes in the rural food environment are associated with dietary intake and nutritional status and to identify appropriate points of intervention and policy opportunities to mitigate the negative and amplify the positive impacts of urbanization on the food environment. In addition, future food environment research should explore novel ways to improve the measurement of spatial accessibility. For example in health systems, spatial accessibility is measured as a combined measure of the density of providers and proximity to providers (e.g., two-step floating catchment area) (McGrail, 2012). Learning from other disciplines and exploring novel methods to better capture access are needed to improve food environment measures (Downs et al., 2024).

## Funding

Funding was provided by the Bill and Melinda Gates Foundation (GH614 and OPP1163259), Johnson & Johnson, the UBS Optimus Foundation, and the U.S. Agency for International Development (Micronutrients for Health Cooperative Agreement HRN-A-00-97-00015-00 and Global Research Activity GHS-A-00-03-00019-00). Under the grant conditions of the Bill and Melinda Gates Foundation, a Creative Commons Attribution 4.0 Generic License has already been assigned to the Author Accepted Manuscript version that might arise from this submission. AB was supported by a Procter and Gamble Fellowship and Tuition Scholarship from the Johns Hopkins Bloomberg School of Public Health Department of International Health. The funders contributed to the trial's design, but played no role in data collection, analysis, or preparation of the manuscript.

## CRediT authorship contribution statement

**Alexandra L. Bellows:** Writing – original draft, Formal analysis, Conceptualization. **Amanda C. Palmer:** Writing – review & editing, Supervision, Conceptualization. **Frank Curriero:** Writing – review & editing, Supervision, Methodology, Formal analysis. **Andrew L. Thorne-Lyman:** Writing – review & editing, Supervision, Methodology. **Abu Ahmed Shamim:** Writing – review & editing, Project administration, Methodology, Data curation. **Saijuddin Shaikh:** Writing – review & editing, Project administration, Methodology, Data curation. **Rezwanul Haque:** Writing – review & editing, Project administration, Methodology, Formal analysis, Data curation. **Hasmot Ali:** Writing – review & editing, Project administration, Methodology, Investigation, Data curation. **Jonathon D. Sugimoto:** Writing – review & editing, Methodology, Formal analysis, Data curation. **Parul Christian:** Writing – review & editing, Methodology, Funding acquisition, Data curation. **Keith P. West:** Writing – review & editing, Project administration, Investigation, Funding acquisition. **Alain B. Labrique:** Writing – review & editing, Methodology, Data curation.

## Declaration of competing interest

All authors declare that they have no known competing financial interests or personal relationships that could have appeared to influence the work reported in this paper.

## Data Availability

Data will be made available on request.
