# Supplementary material for: Changes in urbanicity and household availability of and proximity to food vendors from 2004 to 2020 in a rural district of northwestern Bangladesh
Source: Health Place. 2024 Nov;90:103374. doi: 10.1016/j.healthplace.2024.103374 (PMC11609940; doi:10.1016/j.healthplace.2024.103374)
Supplement: Multimedia component 1 [file mmc1.docx]

**Supplemental Tables:**

| **Supplemental Table 1.** Scoring system and subscores used to define community urbanicity score for n=146 mauzas, by trial cohort | | |
| --- | --- | --- |
| **Domain** | **Variable** | **Scale Scoring** |
|  |  |  |
| Demographic | Population density (people/km^2^)^a^ | 0-750 – 0 points |
|  |  | 750-1000 – 1 point |
|  |  | 1000-1200– 2 points |
|  |  | 1200-1400 – 3 points |
|  |  | 1400-1600 – 4 points |
|  |  | 1600-2000 – 5 points |
|  |  | >2000 – 6 points |
| Economic Activity | % Population involved in agriculture^b^ | 10*(1-Proportion points) |
| Built Environment | Presence of a paved road^c^ | 2 points |
|  | Community electricity access^b^ | 2 points |
|  | % Households with electricity^b^ | 2*Proportion points |
|  | % Households with flush toilet^b^ | 4*Proportion points |
| Communication | % Households with television^b^ | 5*Proportion points |
|  | % Households with mobile phones^b^ | 5*Proportion points |
| Education | % Women with secondary education^b^ | 8*Proportion points |
|  | Presence of a primary school^e^ | 1 point |
|  | Presence of a secondary school^e^ | 1 point |
| **Urbanicity Score** | Mean ± SD |  |
| ^a^ Population density data from the WorldPop database (22). Data were accessed for 2003 (JiVitA-1), 2009 (JiVitA-3), and 2018 (mCARE-II), which corresponded to the midpoint of the date ranges for baseline demographic surveys in the three trials. b Data from trial household demographic surveys. Data for JiVitA-1 were collected from 2001-2006 (n=47,710). Data for JiVitA-3 were collected from 2008-2011 (n=39,362). Data for mCARE-II were collected from 2016-2019 (n=23,809).  c Data on paved roads are from Bangladesh Local Government Engineering (LGED) Road Database (20).  d Data on mobile phones were not available for JiVitA-1 trial.  e Data from study site GPS survey from October 2004 (JiVitA-1), June 2009 (JiVitA-3), and December 2020 (mCARE-II) | | |
